# Long-Term Persistency of Abnormal Heart Rate Variability following Long NICU Stay and Surgery at Birth

**DOI:** 10.1155/2014/121289

**Published:** 2014-02-26

**Authors:** Mélanie Morin, Serge Marchand, Louis Couturier, Sophie Nadeau, Sylvie Lafrenaye

**Affiliations:** ^1^Faculté de Médecine, Université de Sherbrooke, 3001 12e Avenue Nord, Sherbrooke, QC, Canada J1H 5N4; ^2^Département de Chirurgie, Université de Sherbrooke, 3001 12e Avenue Nord, Sherbrooke, QC, Canada J1H 5N4; ^3^Département de Pédiatrie, Centre Hospitalier de l'Université Laval, 2705 Boulevard Laurier, Québec, QC, Canada G1V 4G2; ^4^Département de Pédiatrie, Centre Hospitalier Universitaire de Sherbrooke, 3001 12e Avenue Nord, Sherbrooke, QC, Canada J1H 5N4

## Abstract

Preterm birth is associated with painful procedures during the neonatal intensive care unit (NICU) stay. Full-term newborns can also experience pain, following surgery. These procedures can have long-lasting consequences. It has been shown that children born preterm show pain responses and cardiac alterations. This study aimed to explore the heart rate reactivity to pain in 107 subjects born either preterm or full-term who were between 7 and 25 years old at testing. We also evaluated the effect of pain experienced at birth, as represented by a longer NICU stay, time under ventilation, and surgery at birth. Participants were asked to immerse their right forearm in 10°C water for 2 minutes. Electrocardiograms were recorded at baseline and during the immersion procedure. Full-term subjects showed a stable increase in heart rate throughout the procedure, whereas preterm ones showed a strong increase at the beginning, which decreased over time. Also, preterm and full-term subjects who experienced pain at birth showed higher resting heart rate, stronger sympathetic activity, and lower cardiac vagal activity. Our study demonstrated a long-term impact of a long NICU stay and surgery at birth on cardiac autonomic activity. This could lead to impaired reactions to pain or stress in later life.

## 1. Introduction

In the last decades, advances in knowledge and improvements in treatment options have increased survival rates for preterm infants [1]. However, this success is associated with numerous painful procedures in preterm babies. In the neonatal intensive care unit (NICU), preterm newborns undergo intubations, blood samplings, and many other painful procedures [2]. These procedures occur at a developmental period during which pain transmission mechanisms are well developed [3] but pain modulation mechanisms are still immature [4, 5]. Many studies reported altered pain responses in preterm children [6–12]. Even though changes in heart rate (HR) after a painful procedure are often used to evaluate pain in newborns, none of these studies reported heart-rate variability differences. Cardiac autonomic alterations have, however, been noted for preterm children and adolescents [13, 14]. Higher resting blood pressure has been described in subjects born preterm compared to full-term [13]. Moreover, a recent study revealed that adults born preterm (18 to 24 years old) showed higher resting systolic blood pressure and HR but a lower diastolic blood pressure compared to subjects born full-term [14]. The variability in HR is linked to the sympathovagal balance. No study has yet evaluated if sympathovagal imbalance could explain the higher HR in preterm subjects and if this imbalance could be associated with the pain and stress suffered during NICU stay.

Only a few studies evaluated heart rate changes in reaction to a painful procedure and have yielded contradictory results [9, 15]. The first study by Grunau and colleagues [9] showed that lower gestational age was associated with a dampened cardiac response to pain. Conversely, a later study reported that preterm infants compared to full-term ones showed no differences in heart rate responses to immunization [15]. To our knowledge, there is only one study that evaluated pain-evoked changes in heart rate in preterm compared to full-term older children (7 to 11 years old) during an experimental painful procedure [11]. In that study, after classifying preterm children into two groups according to the extent of pain experienced at birth (low-pain or high-pain, according to their number of painful procedures retrieved from their chart review), an analysis revealed that term-born and low-pain preterm children showed a significant increase in heart rate during the experimental pain test. However, high-pain preterm subjects showed no changes in heart rate, suggesting altered cardiac autonomic adjustments. In the present study, we explored HR reactivity to pain in subjects either born preterm or full-term in a wider age range (from childhood to adulthood). Following the results of Goffaux and colleagues [11], we also evaluated the impact of pain at birth on our results by comparing subjects using the length of stay in the NICU and the time under mechanical ventilation as proxies for the amount of pain suffered at birth in preterm subjects. We also included a group of full-term subjects who underwent cardiac surgery at birth, which is known to be a painful surgery despite the use of analgesics. Subjects born from diabetic mothers were excluded because they usually experience a lot of painful procedures in a short time frame, which would have influenced the correlation between the length of stay and the number of painful procedures.

## 2. Method

### 2.1. Participants

One hundred seven French-speaking subjects aged between 7 and 25 years (mean: 14.6 ± 4.5 years) participated in the study. Healthy control subjects were recruited through local ads. All preterm subjects born at the Centre Hospitalier Universitaire de Sherbrooke (CHUS) or at the Royal Victoria Hospital of Montreal and full-term subjects that underwent surgery at birth at the Montreal Children's Hospital received a letter inviting them to participate in the study. Recruitment was difficult, as only 8% of the subjects contacted by mail sent back a positive answer. All subjects were pain-free at the time of experiment and none of them had taken analgesics or any other medication prior to testing. None of the participants presented any cognitive problems, renal or hepatic failure, cerebral palsy, or epilepsy. The hospitals' review boards approved the study protocol and the experiments took place at the Clinical Research Center of the CHUS, Sherbrooke, Quebec, Canada, and at the Montreal Children's Hospital, Montreal, Quebec, Canada. For subjects under 18 years old who accepted to participate in the study, parents gave their informed consent for their child's participation. Participants 18 years and above signed the consent form before the experiments.

**Figure 1:**
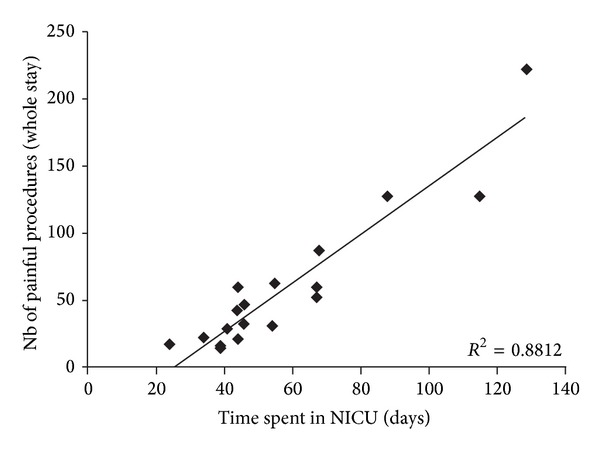
Correlation between the number of days spent in the NICU and the number of painful procedures.

Medical birth files were revised after experimental pain testing by two of the authors (Mélanie Morin and Louis Couturier) to determine the extent of painful procedures experienced at birth for preterm subjects and the type of surgery for full-term participants. Since the information on the exact number of painful procedures experienced at birth was not always available in the birth files, which resulted in missing data for some subjects, we evaluated the number of days in the NICU and under mechanical ventilation. The correlation was high between the number of days spent in the NICU and the number of painful procedures (heel sticks, tracheal suctioning, venipuncture, etc.) (see Figure 1). Therefore, the separation was made according to number of days spent in the NICU. The median number of days under mechanical ventilation, which has also been shown to correlate with the number of painful procedures, was used as well [16]. For the rest of the text, the terms “low-pain” and “high-pain” are used to clearly distinguish our preterm groups. Cardiac surgery occurred in the first days of life in all full-term subjects who underwent surgery (mean: 9.5 ± 8.9 days).

Data was not available for all subjects (*n* = 18) because the medical files of subjects born before 1992 did not explicitly include a detailed report of the painful procedures (e.g., blood sampling). Nonetheless, there is a strong significant positive correlation (*r* spearman = 0.893, *P* < 0.0001) between the number of days spent in the NICU and the number of painful procedures for these subjects. It would be unlikely that the subjects for which the files were not complete show a different correlation.

### 2.2. Cold Pressor Test (CPT)

A cold water bath was used as a test stimulus to cause a prolonged pain sensation. This experimental procedure is commonly used in children to assess pain [11, 17, 18]. Participants immersed their right forearm in circulating cold water maintained at 10°C for two minutes.

### 2.3. Cardiac Activity

Electrocardiogram (ECG) activity was recorded using a standard 3-lead montage sampled at a frequency of 1000 Hz with the Powerlab system and Chart software (AD Instruments, Colorado Springs, CO). ECGs were recorded for two minutes prior to testing (baseline) and during the CPT (immersion). Instantaneous RR intervals were obtained from the ECG waveform with a peak detection algorithm to detect successive R-waves. HR variability analyses in the frequency domain were done subsequently. Fast Fourier transforms were used to calculate the power spectral density of HR oscillations (window length: 1024). The FFT method can provide a noisy estimator of the power spectrum. This is why we used it in combination with Welch's method. This allows us to achieve a consistent estimator by averaging periodogram from overlapping intervals [19]. In our case, the overlap was 1/2 or 50%. The analysis of the HRV was made on a 2-minute window, before and during the painful procedure. Low frequency (LF: 0.04–0.15 Hz) and high frequency (HF: 0.15–0.4 Hz) components were analyzed. These components can be used as an indication of cardiac autonomic nervous system activity [20, 21]. LF is an indicator of sympathetic activity [22, 23] and HF an indicator of cardiac parasympathetic activity [21, 24]. Normalized HF and LF values were computed and analyzed in our study. Also, cardiac sympathovagal balance can be evaluated with the LF/HF ratio. HR reactivity was calculated as the percentage change in HR between immersion and baseline ((HR_CPT_ − HR_baseline_)/HR_baseline_). We reported HR reactivity for the mean of every 15 seconds or as a mean of the entire 2-minute immersion.

### 2.4. Procedure

Demographic information was collected prior to testing for each participant. Pediatric ECG electrodes were first installed and baseline ECG was obtained as the participants were asked to relax. Participants were then asked to immerse their right arm in cold water, while ECG was recorded.

### 2.5. Statistical Analysis

Descriptive statistics are presented as mean ± standard deviation (SD). After checking for assumptions of normality and homogeneity of variances, Kruskal-Wallis tests and 2 × 2 ANCOVAs were used. Bonferroni post hoc analyses were used to evaluate the differences between the specific groups. For the repeated measures of HR during the cold water test, we used the mixed procedure of the SAS System for Windows, version 9.1 (SAS Institute, Cary, NC, USA). The fixed part of the model contains a linear and a quadratic effect of time and the interaction between each of those factors and the group (preterm or full-term) to test if the linear and quadratic effect changes between groups. To account for the correlated residuals within subjects, a repeated statement with an unstructured covariance structure was used on time. The unstructured covariance structure gave the best Akaike Information Criterion (AIC) compared to other structures (e.g., AR(1) and CS) and therefore was used for the analysis. *P* < 0.05 (two-tailed) was considered statistically significant.

## 3. Results

### 3.1. Heart Rate Changes

**Figure 2:**
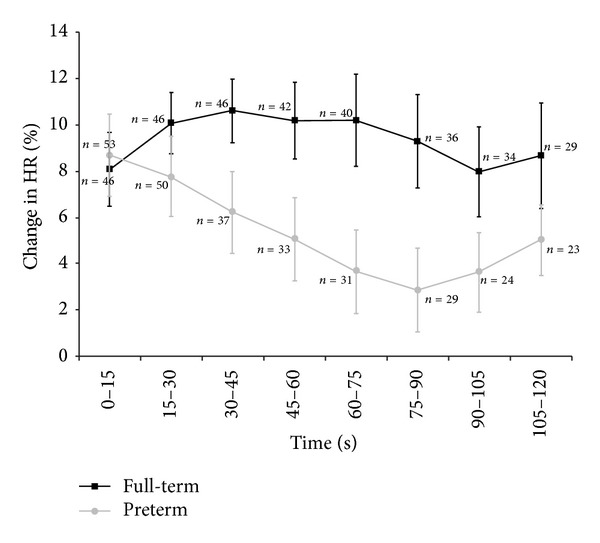
Average percentage change in heart rate from baseline (SEM) during the CPT for 15 s intervals.

There were no significant group differences (preterm versus full-term) in resting heart rate (*t* = −1.43, *P* = 0.16). Both groups had a comparable change in HR at the beginning (first 15 seconds) of the procedure (*t* = −0.25, *P* = 0.80). However, an analysis of the change in HR during the CPT, evaluated every 15 seconds, showed that full-term subjects experienced a more stable increase in HR during the immersion compared to the preterm participants. Preterm subjects had a strong increase at the beginning of the immersion and HR slowly decreased over time. An analysis revealed that the birth type (preterm versus full-term) had an impact on the course of the percentage change during the procedure (quadratic relationship: *F* = 5.76, *P* = 0.018), with age having a significant effect (*F* = 6.10, *P* = 0.015) when entered as a covariate (Figure 2 and Table 1).

There was a different quadratic relationship between the percentage change and time in full-term compared to preterm subjects. The full-term group shows an increase of their percent change in HR over time followed by a decreased (concave function) while the preterm group shows an opposite effect. They show a high increase at the beginning that instantly decreases (convex function).

### 3.2. Impact of Pain Experienced at Birth

Further analyses were made to verify whether pain experienced at birth had an impact on mean percentage change in HR. The subjects were divided into 4 groups (full-term, full-term with surgery, low-pain preterm, and high-pain preterm) according to their type of birth and the length of stay in the NICU and number of days under mechanical ventilation, as proxies for the pain suffered at birth. There were significant differences for GA, birth weight, 5 min APGAR, days under mechanical ventilation, and days spent in NICU between the groups (Kruskal-Wallis test: all *P*s < 0.05) (Table 2). Bonferroni post hoc analysis showed that both preterm groups had significantly lower birth weights and gestational ages than full-term surgery subjects (all *P*s < 0.03). The preterm groups also differed from one another for those variables (both *P*s < 0.001). The high-pain preterm group had more days of ventilation (*P* < 0.001) and spent more days in the NICU (*P* < 0.001) than the low-pain preterm subjects. The full-term surgery and high-pain preterm groups differed only in GA, birth weight, and time spent in NICU (all *P*s < 0.001) (Table 2).

A 2 × 2 ANCOVA was used to determine the impact of type of birth and pain experienced at birth on mean baseline HR with age at testing as a covariate. Pain experienced at birth had a significant impact (*P* = 0.041) and age at testing was a significant factor (*P* < 0.001). Full-term surgery and high-pain preterm groups showed significant higher resting HR (Tables 3 and 4).

A 2 × 2 ANCOVA also revealed that the pain experienced at birth had a significant impact on the LF (*P* = 0.013), HF (*P* = 0.003), and the ln of the ratio LF/HF (*P* = 0.004) (see Tables 3 and 4). In order to be able to use a parametric test, the ln of the ratio was used because the data was not normally distributed for this variable. Baseline values of ECG activity show that the full-term surgery and high-pain preterm groups have a stronger sympathetic activity (LF) and a lower parasympathetic (HF) cardiac activity at rest. They also show a sympathovagal imbalance (higher LF/HF) compared to the groups who did not experience pain at birth.

A 2 × 2 ANCOVA revealed that the pain experienced at birth showed a significant impact on the mean percentage change in HR (*P* = 0.006), with age at testing being a significant covariate (*P* = 0.027) (Tables 3 and 4). Full-term surgery and high-pain preterm groups showed a significantly lower percentage change in HR over the 2-minute immersion.

## 4. Discussion

It has previously been suggested that numerous painful interventions in the neonatal period could compromise the cardiovascular autonomic responses typically observed during painful stimulations in later life [11]. In this study, we explored the heart rate reactivity to pain in children, adolescents, and young adults either born preterm or full-term, years after they underwent neonatal pain. To evaluate the impact of pain at birth (determined by the number of days in the NICU and the number of mechanical ventilation), we compared them to a group of subjects born full-term without surgery and a group of preterm participants who suffered only a few painful procedures at birth. In these four groups of patients, we observed significant heart rate differences.

Despite a comparable percentage change of HR in the first 15 seconds of the CPT, preterm and full-term subjects differed in the evolution of their HR response. Full-term subjects had a mean increase of 8.2 ± 1.2 bpm that was relatively stable over time, compared to preterm subjects who started with a mean increase of 8.7 ± 1.6 bpm that decreased over time. A previous study showed that only preterm children (7–11 years old) who experienced many painful interventions at birth exhibited a complete absence of rise in HR during CPT [11].

Similar results are found when comparing mean percentage change in HR. In fact, high-pain preterm subjects show a lower mean percentage change in their HR. In addition, we found that full-term subjects with surgery also showed a lower mean percentage change in HR. These findings suggest that having suffered intense pain at birth regardless of gestational age alters heart rate changes to experimental pain. To our knowledge, no other study has clearly exposed this relationship. Previous studies only compared preterm to full-term subjects. Preterm infants who experienced a higher number of painful procedures following birth showed less autonomic reactivity [6, 8]. They also displayed a faster cardiac recovery after a painful stimulus compared to term-born infants [25]. This is consistent with what we observed in our preterm group even at adult age, suggesting important long-term consequences. In fact, Grunau and colleagues [25] suggested that differences between preterm and full-term children appeared to increase over time.

A higher baseline HR was observed in subjects who underwent painful procedures at birth, when age at testing was entered as a covariate in the analysis. This difference is not found if we compare full-term to preterm birth, suggesting that the pain suffered is the cause of the altered HR. This is consistent with previous studies that found that the high-pain preterm group showed a higher resting HR, suggesting a state of chronic arousal [11, 25, 26]. The impact of age at testing found in our study is probably related to the wide age range (7–25 years old) of the subjects recruited. It has been previously described that heart rate variability as well as resting HR are age-dependant [27–29]. This is particularly important, because it has been shown that a higher resting HR is linked to an increased risk factor for mortality from coronary disease, cardiovascular diseases, and cancer [30, 31].

We also found important autonomic reactivity differences in our groups. In fact, full-term surgery and high-pain preterm participants showed a stronger sympathetic activity (LF) and a lower cardiac vagal activity (HF) at rest, suggesting again a state of chronic arousal. Our study confirmed a sympathovagal imbalance in subjects who experienced pain at birth (either born preterm or full-term). This sympathovagal imbalance has also been found in subjects with migraine and alterations in autonomic nervous system function and is thought to contribute to the development of symptoms in these patients [32]. Because HRV alterations are associated with stroke [33], cardiac morbidities [34], chronic pain [35], and an increase in mortality [36], future epidemiological studies should investigate if these subjects are at greater risk of morbidities in adulthood.

However, some limitations should be taken into account concerning our study. First, not all subjects were able to complete the entire 2-minute CPT procedure (47%). A minimum recording of two minutes of the heart rate has been shown to be necessary to perform a short-term HRV analysis [20]. Consequently, for some subjects the LF, HF, and LF/HF ratio could not be computed during the CPT (full-term: *n* = 16, low-pain preterm: *n* = 15, high-pain preterm: *n* = 16, and full-term surgery: *n* = 3). Even when 2-minute recordings are used, the stationarity of the signal may be not respected and the Fast Fourier Transform algorithm used to analyse the HRV may lead to misleading results. Therefore, we need to be cautious in the interpretation of the results. Also, we used the length of stay in the NICU and the number of days under mechanical ventilation as proxies for the extent of pain experienced at birth, because the exact number of painful procedures was not always available due to archive procedures. Nonetheless, we were able to determine that the length of stay was in fact correlated to the number of painful procedures experienced at birth in the preterm group. However, other confounding factors like maternal separation or stress could be associated with a longer stay in the NICU and a higher number of days under mechanical ventilation and have an impact on the cardiac pain response. The health of the infant at birth, which could have also increased the NICU stay, as well as stress caused by painful procedures and maternal separation [37], could have impacted directly their HR or HRV responses and therefore explain part of the relationship. It would be interesting to try to evaluate the impact of those factors in this relationship in future studies. Also, in the full-term surgery group, all subjects underwent a cardiac surgery at birth, which in itself could have an impact on later cardiac responses to pain. Further studies would be needed to examine the extent of this impact.

## 5. Conclusion

Clinically, our results have important implications because they demonstrate an impact of the length of stay in the NICU and the number of days under mechanical ventilation (proxies used to evaluate the number of painful and stressful procedures experienced at birth) on resting and stressed autonomic cardiac activity years after the stay in the NICU. Subjects with prior pain exposure, either born preterm or full-term, showed a higher baseline HR, a stronger sympathetic activity, and a lower cardiac vagal activity even at adulthood. This study adds to the prevailing literature in the sense that the impact on heart rate comes from pain experienced at birth (represented by a longer NICU stay and time under mechanical ventilation in preterm or surgery in full-term subjects), independently of the birth status (preterm versus full-term), and is still found in young adulthood. This could lead to impaired reactions to pain or stress in later life. It is important to consider the long-term impact of pain at birth, even in the presence of some analgesia in full-term neonates who underwent surgery. We showed that the regulation of the cardiac autonomic system is still altered years after all procedural pain is over. Conclusions of our study show the importance of providing better analgesic care for the neonates and limiting as much as possible painful medical procedures during this critical time.

## Figures and Tables

**Table 1:** Unstructured covariance structure for percentage change in HR.

	df	*F *	*P *
Time	1	0.15	0.697
Group	1	1.07	0.303
Time × group	1	6.92	0.010
Time × time	1	0.35	0.558
Time × time × group	1	5.76	0.018
Age	1	6.10	0.015

**Table 2:** Birth characteristics (median (25–75)).

	Full-term (*n* = 46)	Full-term surgery (*n* = 8)	Low-pain preterm (*n* = 29)	High-pain preterm (*n* = 24)	*P* Kruskal-Wallis
Age at testing	15 (11–17)	15 (13–17)	14 (10–17)	16 (10–19)	0.756
Gestational age (weeks)	—	40 (39-40)	32 (31–33)	28 (27–31)	<0.001
Birth weight (g)	—	3380 (2862–3935)	1602.5 (1405–2081)	1220 (920–1350)	<0.001
5 min APGAR	—	9 (9-9)	8 (7–9)	8 (6–9)	0.005
Days under mechanical ventilation	—	12 (3–15)	0 (0-1)	28 (6–44)	<0.001
Days spent in NICU	—	20 (14–33)	35 (18–46)	77 (51–102)	<0.001

**Table 3:** Heart rate variability (mean ± SD).

	Full-term (*n* = 46)	Full-term surgery (*n* = 8)	Low-pain preterm (*n* = 29)	High-pain preterm (*n* = 24)
Baseline				
Heart rate	78.2 (12.5)	84.5 (12.7)	80.2 (14.3)	84.5 (15.1)
LF (nu)	46.9 (19.9)	61.3 (17.5)	49.8 (19.9)	58.8 (17.4)
HF (nu)	43.8 (18.5)	27.7 (10.8)	42.5 (19.0)	32.7 (14.5)
LF/HF*	1.2 (0.4–1.8)	2.0 (1.4–3.2)	1.4 (1.4–2.6)	1.9 (1.3–3.8)

% change in HR	9.1 (8.8)	2.9 (9.6)	8.8 (9.9)	3.9 (9.0)

*Since the LF/HF ratio was not normally distributed, the median (25–75) is shown in the table.

**Table 4:** ANCOVA results.

	HR (bpm)			LF (nu)			HF (nu)			ln (LF/HF ratio)			% change in HR		
	df	*F *	*P *	df	*F *	*P *	df	*F *	*P *	df	*F *	*P *	df	*F *	*P *
Type of birth	1	0.135	0.715	1	<0.001	0.985	1	0.223	0.638	1	0.131	0.718	1	0.039	0.844
Pain at birth	1	4.275	**0.041**	1	6.414	**0.013**	1	9.466	**0.003**	1	8.448	**0.004**	1	7.856	**0.006**
Type of birth × pain	1	0.059	0.808	1	0.492	0.485	1	0.672	0.414	1	0.756	0.387	1	0.041	0.841
Age	1	19.971	**<0.001**	1	16.675	**<0.001**	1	4.492	**0.036**	1	9.711	**0.002**	1	5.033	**0.027**
